# Feasibility and safety of reduced-port video-assisted thoracoscopic surgery using a needle scope for pulmonary lobectomy– retrospective study

**DOI:** 10.1016/j.amsu.2019.07.027

**Published:** 2019-07-26

**Authors:** Masato Aragaki, Kichizo Kaga, Yasuhiro Hida, Tatsuya Kato, Yoshiro Matsui

**Affiliations:** Department of Cardiovascular and Thoracic Surgery, Hokkaido University Graduate School of Medicine, West-7, North-15, Kita-ku, Sapporo, Hokkaido, 060-8638, Japan

**Keywords:** Congenital pulmonary cystic disease, One-window and puncture method, Reduced port surgery, Video-assisted thoracoscopic surgery

## Abstract

**Background:**

This study aimed to determine the usefulness and limitations of videoassisted thoracoscopic (VATS) lobectomy using one-window and puncture method (1WPM).

**Methods:**

This study involved 14 patients who underwent lobectomy using the 1WPM at our institute from 2008 to 2017.

**Results:**

The study patients comprised of 3 men and 11 women with a median age of 10.5 years (range, 0-72 years). There were eight cases in children younger than 18 years old and the youngest patient was 9 days old. The diagnoses were congenital pulmonary cystic disease (n = 7), primary lung cancer (n = 4), metastatic lung tumor (n = 1), and others (n = 2). The 1WPM was successful in 9 of 14 patients (64.3%) and, in 5 cases (35.7%), needed conversion to either two-window method (TWM) using additional port (n = 3) or open thoracotomy (n = 2). The causes for conversion were need for additional bronchoplasty or lymph node dissection (n = 3), failure of one-lung ventilation (n = 1), and presence of a small thoracic cavity that made the procedure extremely difficult (n = 1). In the group that was successfully treated with 1WPM, the median values were as follows: operation time, 193 min (range, 112-480 min); blood loss, 0 ml (range, 0-90 ml); drainage duration, 1 day (range, 1-4 days); and postoperative hospital stay, 7 days (range, 4-13 days).

**Conclusions:**

Lobectomy by 1WPM can be safely performed and has good postoperative course and this procedure can be applicable and effective in small infants.

## Highlights

• This study describes the novel VATS lobectomy method: the one-window and puncture method (1WPM).
• 1WPM is performed using 2–3 cm single incisions and a needle scope for intrathoracic access from other points.
• This retrospective study involved 14 patients who underwent lobectomy using the 1WPM.
• VATS lobectomy using 1WPM can be safely applied from adult cases to new born cases.

## 1 Introduction

Since its initial introduction in the early 1990s [1,2], video-assisted thoracoscopic surgery (VATS) has become the standard procedure for pulmonary lobectomy. Recently, surgeries minimizing invasiveness have been developed including reduced-port surgery, which includes uniportal incision [[3], [4], [5], [6], [7]], two-port access [[8], [9], [10]], and needlescopic VATS [11]. However, there are few reports discussing limitations and indications for each procedure [12,13].

We use two standard approaches in VATS for lobectomy in lung disease: one approach, the two-window method (TWM), which uses two ports along the posterolateral incision line [8], or the one-window and puncture method (1WPM), which uses one-port access and a needle scope [11].

This study focused on the 1WPM during VATS for pulmonary lobectomy to determine its usefulness and limitations, and evaluated its feasibility and safety.

## 2 Methods

### 2.1 Patients

This retrospective, single-center study included data from 14 subjects who underwent pulmonary lobectomy by VATS using the 1WPM at our institute between 2008 and 2017. Written informed consent was obtained from all patients. Patients who did not require lymph node dissection and met provisional indications were selected for the 1WPM. Conventional TWM, following our standard institutional approach, or standard thoracotomy were used when the 1WPM was not feasible and these patients were excluded from the study. We examined sbackground factors, including age, sex, height, body weight, BMI, laterality of surgery, surgical objective, target, location, and operative procedure to determine the appropriateness of provisional indications. The information was retrieved from our hospital database and the study did not require approval by any ethics boards. The study was reported in line with the STROCSS criteria [12]. This study was registered with Researchregistry.com (ID no: researchregistry 4882).

### 2.2 Statistical analyses

Descriptive statistics were reported as median (range), and categorical data were reported as percentages (%). Statistical analyses were performed using JMP software (SAS Institute, Cary, North Carolina, USA).

### 2.3 Surgical technique

**Fig. 1:**
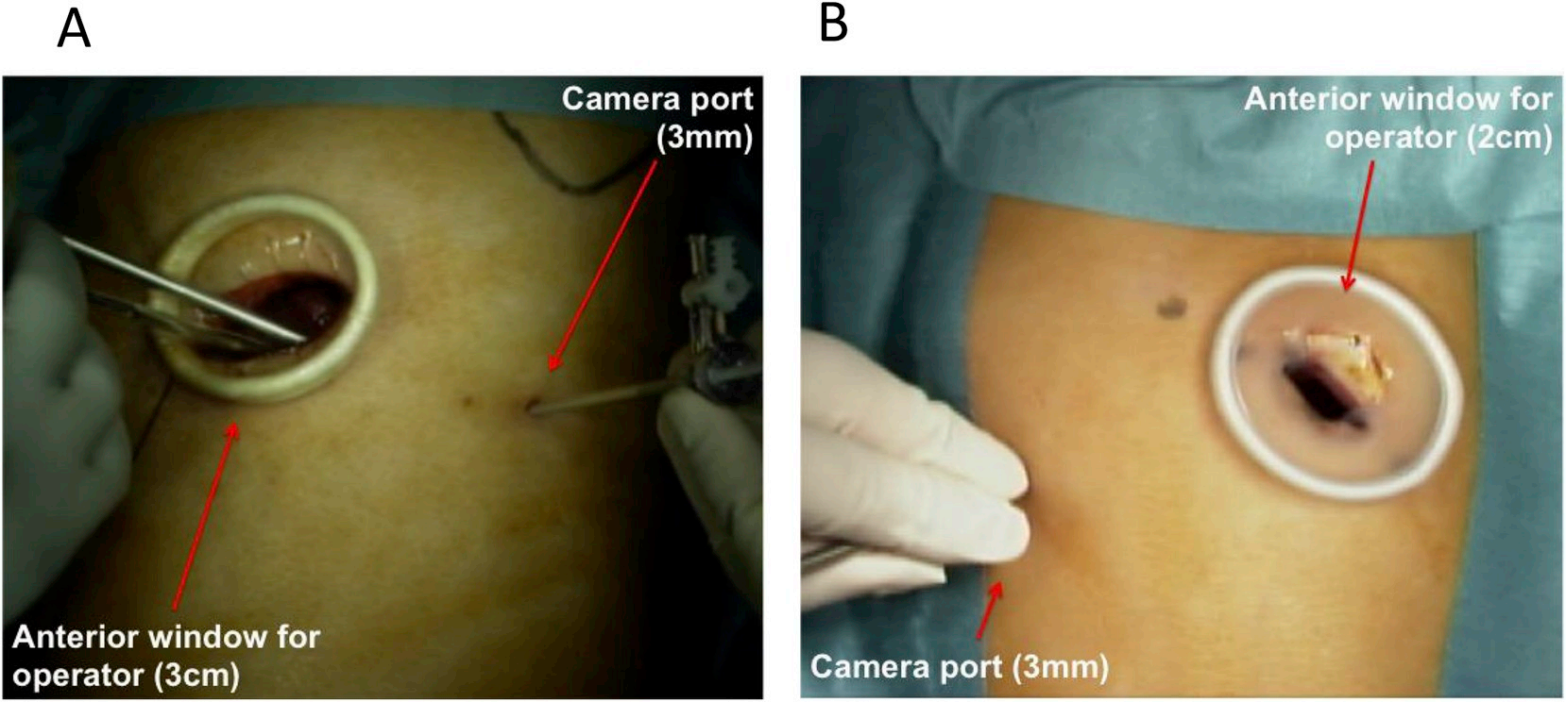
Skin incision of the 1WPM technique (A) Left lower lobectomy in an adult patient; (B) right lower lobectomy in a pediatric patient. 1WPM.

**Fig. 2:**
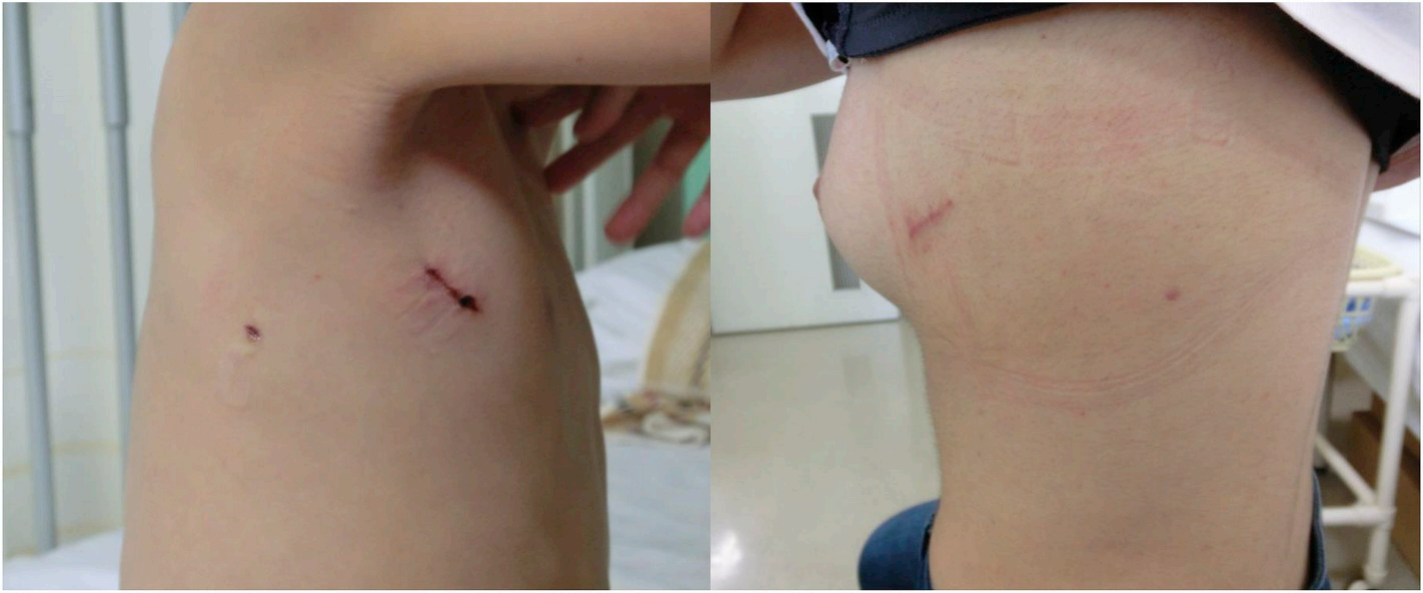
Postoperative results of the 1WPM 1WPM.

**Fig. 3:**
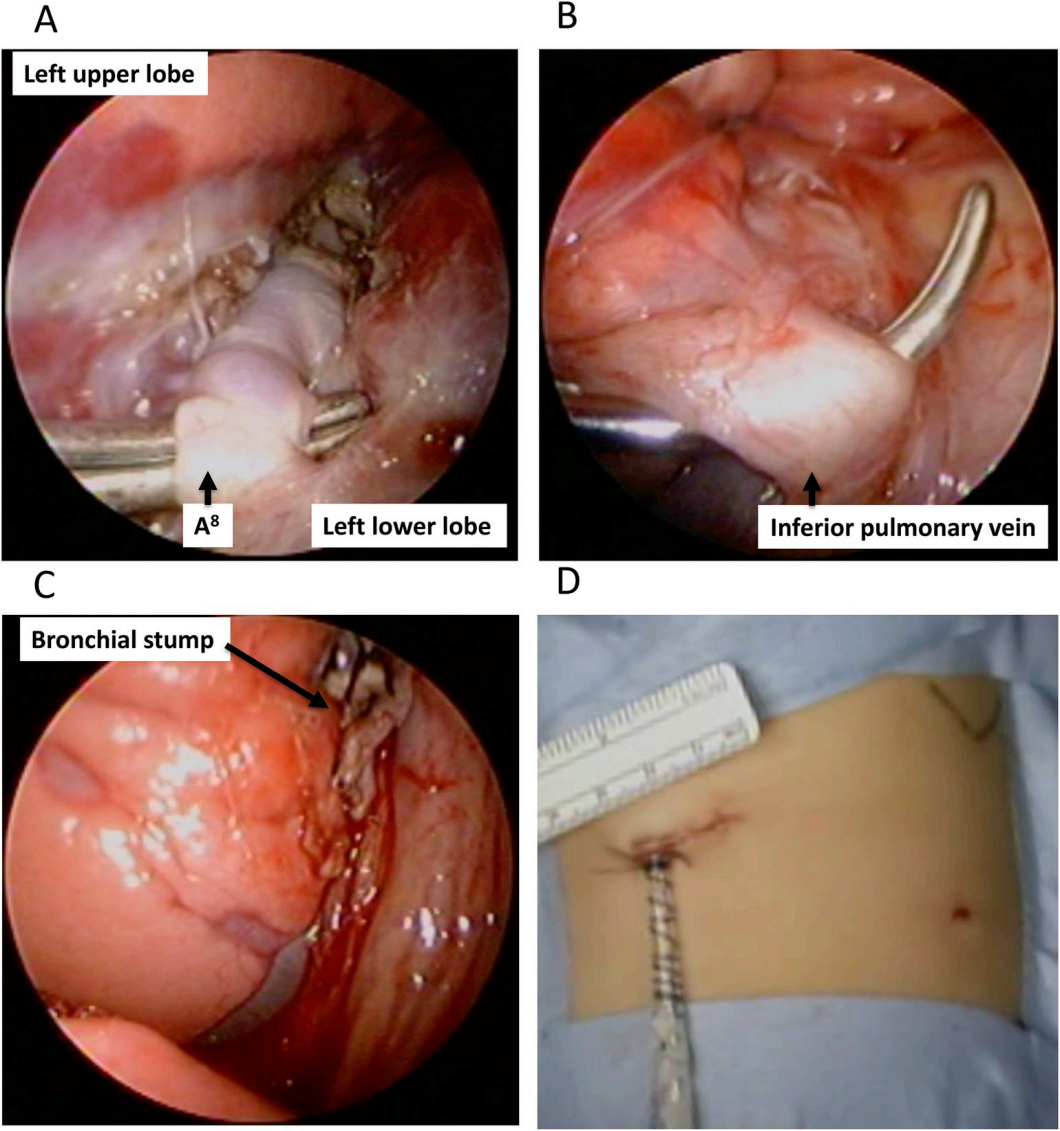
Intraoperative image of the 1WPM for a left lower lobectomy. (A)Encircling of the pulmonary artery (A^8^); (B) Encircling of inferior pulmonary vein; (C) View after bronchial stump cutting; (D) Surgical wound immediately after surgery. 1WPM.

Surgery was performed under general anesthesia using one-lung ventilation. For the 1WPM, a 2–4 cm incision was made on the anterior or posterior midaxillary line of the fifth or sixth intercostal space (Fig. 1, Fig. 2). An Alexis wound retractor XSTM (Applied Medical, Rancho Santa Margarita, CA, USA) or LAPPROTECTORTM mini-mini (Hakko medical, Tokyo, Japan) was used during thoracotomy. A 30° rigid scope measuring 3 mm or 5 mm in diameter (Olympus Medical Science, Tokyo, Japan) permitted intrathoracic observation to confirm the indications for the 1WPM. An additional puncture was made for the needle scope if insertion through the initial incision site was insufficient. Surgical procedures were occasionally assisted by the pulling method using silk or needle forceps through additional puncture points (Fig. 3). The resected specimen was placed in a plastic bag and extracted. After confirming the absence of air leakage using a sealing test, 12–20 Fr drainage tubes were inserted to complete the surgery.

## 3 Results

Patient characteristics and clinical findings are shown in Table 1. The cohort of 14 patients included 3 men and 11 women with a median age of 10.5 years (range, 0–72 years). At the time of surgery, the median height was 124.5 cm (range, 48–166 cm) and the median weight was 30.5 kg (range, 2.6–76.9 kg). The diagnoses were congenital pulmonary cystic disease (n = 7), primary lung cancer (n = 4), metastatic lung tumor (n = 1), pulmonary sclerosing hemangioma (n = 1), and non-tuberculous mycobacterial infection (n = 1).

**Table 1:** Patient characteristics.

Characteristic	Number
Number of patients	
Sex	
Male	3
Female	11
Age (years)	
Median (Range)	10.5 (0–72)
＜18 years	8
≧18 years, ＜40 years	1
≧40 years	5
Height (cm)	
Median (Range)	124.5 (48–166)
Weight (kg)	
Median (Range)	30.5 (2.6–76.9)
Disease	
Congenital pulmonary airway malfunctions	7
Primary lung cancer	4
Metastatic lung tumor	1
Pulmonary sclerosing hemangioma	1
Nontuberculous mycobacterial infection	1

The surgical procedures were middle lobectomy (n = 5), left lower lobectomy (n = 4), right lower lobectomy (n = 3), and left upper lobectomy (n = 2) (Table 2). The median operative time was 199.5 min (range, 112–507 min) with an estimated blood loss of 0 ml (range, 0–90 ml). The median duration of drainage was 1.5 days (range, 1–7 days), and the median length of hospitalization was 7 days (range, 4–20 days). The 1WPM was completed in 9 cases (64.3%). Five cases (35.7%) required conversion to TWM or thoracotomy (Table 3). Three patients required additional bronchoplasty or lymph node dissection. In two patients, unexpected procedural conversion was related to failure of one-lung ventilation or a small thoracic cavity that made the procedure difficult. No other patients developed major complications.

**Table 2:** Operation procedure.

Case	Lesion	operation time (min)	bleeding (ml)	Conversion	Drainage (days)	hospital stay (days)
1	RLL	193	0	-	4	13
2	RLL	142	0	-	1	4
3	RML	195	50	-	1	4
4	RML	204	20	Conversion	1	7
5	RML	189	0	-	1	7
6	LLL	162	0	-	1	6
7	RLL	480	90	-	2	9
8	LLL	316	0	Conversion	2	7
9	LUL	507	0	Conversion	3	20
10	RML	414	0	-	2	11
11	LUL	507	50	Conversion	1	7
12	LLL	441	30	Conversion	1	7
13	RML	139	0	-	2	5
14	LLL	112	0	-	2	5
Median		199.5	0		1.5	7

**Table 3:** Characteristics of conversion cases.

Case	Age, years	Sex	Height, cm	Weight, kg	Diagnosis	Lesion	operation time, min	bleeding, ml	Conversion type	Reasons for conversion
4	58	M	166	76.9	metastatic lung tumor	RML	204	20	Thoracotomy	need for bronchoplasty
8	63	F	159	50.7	primary lung cancer	LLL	316	0	TWM	need for lymph node dissection
9	0(9days)	F	48	2.6	CPAM	LUL	507	0	Thoracotomy	a small thoracic cavity
11	5	M	99	13.4	CPAM	LUL	507	50	TWM	failure of one lung ventilation
12	24	F	148	47.7	primary lung cancer	LLL	441	30	TWM	need for lymph node dissection

## 4 Discussion

VATS for pulmonary lobectomy was conventionally performed using three or more ports. However, reduced-port surgeries including uniportal VATS and 1WPM have gained interest worldwide. However, these reduced-port procedures are technically demanding, and have issues, such as learning curve, safety, adaptation, and limitations [6,7,[13], [14], [15], [16], [17]]. We have used reduced-port surgery by TWM for pulmonary lobectomy since 2007. With the aim of further decreasing invasiveness, we selected cases from 2008 to 2017 and performed pulmonary lobectomy by the 1WPM.

In this study, it was not feasible to compare the outcomes of pulmonary lobectomy between the 1WPM and other procedures because no patients underwent the conventional method without lymph node dissection. The operative time, blood loss, drainage time, length of hospitalization, and postoperative complications in this study were similar to those reported for the conventional methods and were generally acceptable [6,7,[13], [14], [15]]. The rate of conversion from uniportal VATS lobectomy to open surgery is reported to range from 2% to 23% [6,7,18,19]. In this study, the conversion rate was 35.7% largely due to the necessity for additional procedures such as bronchoplasty and lymph node dissection. Two cases, both of which were pediatric patients, were converted due difficulties during surgery. Therefore, it is necessary to consider not only technical reasons, but also factors such as small physique. Although the number of cases in this study was small, none required emergency thoracotomy due to complications. Overall, these data suggest that surgery by 1WPM can be performed safely.

Reduced-port surgery is important to not only reduce wound pain, blood loss, and operation time, but to also to potentially increase curability. Improvements in long-term results, including recurrence rate of malignant tumors are necessary, but have yet to be analyzed in this context. Lymph node dissection using the 1WPM is technically possible, but since it has not been confirmed whether lymph node dissection during reduced-port surgery effects the survival rate of patients with malignant tumors, it has not been adopted in clinical practice. We believe that medium-to long-term observations after reduced-port surgery with lymph node dissection are needed to evaluate the effects on survival rate of patients with malignant tumors before implementation in the clinic.

Both 1WPM and uniportal VATS are included in the category of reduced-port surgery, but there are several differences between the two. Uniportal VATS entails insertion of both the camera and forceps from one direction, requiring ingenuity in creating the visual field and preparation of special instruments. The 1WPM is performed with three-port VATS or the TWM instrument, eliminating the need to prepare special instruments, which has the merit of being easy to introduce to other facilities.

Second, a 3-mm port can be inserted at the optimal position without limit and serve as a camera for confirmation of the surgical field in the 1WPM. Surgical invasiveness with the 3-mm port is minor because the wound does not need suturing, produces minimal scarring, and minimizes pain. Moreover, the necessary incision length for extraction of the resected specimen is only 35 mm, and the total incision length is less than 40 mm. In children who have smaller lungs, this incision length is approximately 25 mm.

The applicability of the 1WPM for pediatric patients, in whom thoracoscopic surgery is technically difficult due to their smaller physique and working space, is also an advantage. Small size makes VATS lobectomy difficult in children and often requires a special approach; therefore, this operation is performed at only a few facilities [20,21]. Since the surgical instruments for both procedures are the same, surgeons can learn the technique of normal VATS lobectomy before doing the 1WPM in adults. The 1WPM can then be applied to pediatric cases once surgeons become proficient.

However, the applicability of 1WPM in all pediatric cases remains unknown. In this study, all cases of lobectomy by the 1WPM in children required conversion to thoracotomy. This indicates potential limitations in the applicability of the 1WPM in pediatric patients. Limitations based on physique remain an important consideration due to the infrequent use of the 1WPM. Further studies comparing populations with differences in physique are needed to address this gap.

The retrospective design, absence of comparative subjects, and the small number of cases limited this study. In addition, we were not able to compare the different surgical methods under similar conditions. Since sublobar resection for small-lung cancers is widely performed, there are few cases of lobectomy without lymph node dissection. Further investigation with more cases is necessary.

## 5 Conclusion

In conclusion, for patients with lung disease, 1WPM lobectomy can be performed appropriately in selected cases, with a 35.7% conversion rate to conventional VATS or standard thoracotomy. It was a safe surgical procedure and no cases required urgent thoracotomy. In addition, pulmonary lobectomy by 1WPM can be applied to children and is considered to be a safe procedure that can be learned step by step. However, because of the possible limitations in applicability in neonatal cases with small physique, the indications and suitability of this procedure are probably selective.

## Consent

Written informed consent was obtained from the patients for publication. A copy of the written consent form is available for review by the Editor-in-Chief of this journal upon request.

## Provenance and peer review

Not commissioned, externally peer reviewed.

## Ethical approval

A retrospective study that does not require ethical approval.

## Funding

The authors received no financial support for the research, authorship, and/or publication of this article.

## Author contribution

M.A., Y.H., T.K., and K.K. performed the surgical operations. M.A. wrote the manuscript with support from K.K., Y.H., and T.K. All authors discussed the results and contributed to the final manuscript.

## Conflicts of interest

The authors have no conflicts of interest to declare.

## Research registry UIN

Researchregistry4882.

## Guarantor

Dr. Kichizo Kaga M.D., PhD.

Department of Cardiovascular and Thoracic Surgery, Hokkaido University Faculty of Medicine, West-7, North-15, Kita-ku, Sapporo, Hokkaido, 060–8638, Japan.
